# Prenatal Isolated Congenital Diaphragmatic Hernia: A Rare Clinical Presentation of a *GATA4* Pathogenic Variant

**DOI:** 10.1177/10935266251381440

**Published:** 2025-10-16

**Authors:** Nea Tulonen, Jussi Tallus, Heidi Kaprio, Jukka Laine, Mirjami Mattila, Maria Haanpää, Sini Keskinen

**Affiliations:** 1 Tyks Laboratories, Genomics, Clinical Genetics, TYKS Turku University Hospital, Finland; 2 University of Turku, Finland; 3 Department of Radiology, TYKS Turku University Hospital, Finland; 4 Department of Pathology, TYKS Turku University Hospital, Finland; 5 Department of Obstetrics and Gynecology, TYKS Turku University Hospital, Finland

**Keywords:** GATA4, diaphragmatic hernia, prenatal, fetal

## Abstract

Congenital diaphragmatic hernia is a genetically heterogeneous condition with a developmental defect in the diaphragm. The *GATA4* gene is essential for fetal heart development, and pathogenic *GATA4* variants are a known cause of structural congenital heart diseases. Haploinsufficiency of GATA4 is also associated with diaphragmatic hernia. Pathogenic *GATA4* sequence variants with isolated diaphragmatic hernia in the absence of congenital heart defects are extremely rare. Our report expands the phenotypic spectrum related to *GATA4*.

We report a fetus with a prenatal isolated diaphragmatic hernia detected during a routine screening ultrasound. An autopsy of the fetus confirmed a large isolated posterolateral hernia, which affected the left lung volume significantly. Clinical exome sequencing revealed a novel heterozygous nonsense variant c.826C>T,p.(Gln276*) in the *GATA4* gene, which was predicted to cause haploinsufficiency. The variant occurred *de novo* and was classified as pathogenic.

The report presents a detailed clinical description of the fetus with ultrasound, MRI, and post-mortem pictures of a rare prenatal isolated diaphragmatic hernia related to a novel pathogenic *GATA4* sequence variant. Prenatal ultrasound screening with further investigation by MRI and a comprehensive gene panel holds a key role in determining the prognosis of a fetus with a diaphragmatic hernia.

## Introduction

Congenital diaphragmatic hernia (CDH) is estimated to occur in 2–4/10 000 pregnancies.^1^ It is a genetically heterogeneous condition, with even the most common underlying causes accounting for only a few percent each of all CDH incidences.^2^ CDH may be associated with chromosomal aneuploidies, copy number variants, or monogenic disorders,^2,3^ and 40–60% of cases are part of syndromic manifestations.^2^ The defining feature of CDH is a developmental defect in the diaphragm, most commonly located in the left posterolateral region (Bochdalek hernia).

While ultrasound is the primary diagnostic modality in prenatal settings, fetal MRI is used for further assessment of a hernia, screening for additional anomalies, and lung volumetric measurements.^3^ Prenatal detection allows for parental counselling, planning of delivery and postnatal care, and even prenatal interventions such as tracheal occlusion.

Clinical severity of CDH is variable, with mortality after birth mostly dependent on the degree of lung hypoplasia. Lung hypoplasia is caused by the abdominal organs herniating into the thoracic cavity and can be quantified by lung area to head circumference ratio with ultrasound or lung volumetry with MRI. These measurements can be used to evaluate the probability of postnatal survival and extracorporeal membrane oxygenation (ECMO) treatment requirement.^4^

*GATA4* (GATA-binding protein 4) encodes a zinc finger transcription factor, which binds GATA motifs of several genes (OMIM:*600576). The *GATA* gene family is involved in multiple embryogenic events.^5^ *GATA4*, *GATA5* and *GATA6* cooperate in cardiogenesis and are critical in the regulation of fetal cardiac cell specification and heart formation.^6^ Diseases linked to *GATA4* include atrial septal defect (ASD), atrioventricular septal defect (AVSD), ventricular septal defect (VSD) and tetralogy of Fallot (OMIM:*600576).

Notably, *GATA4* has a crucial role in the developing diaphragm, and its reduced expression has been linked to CDH.^7^ The chromosome 8p23.1 microdeletion encompassing *GATA4* is a well-recognised cause of congenital heart defects and diaphragmatic hernia, contributing to 3-5% of CDH cases.^2,8^ *GATA4* is considered the candidate gene for CDH in recurrent 8p23.1 microdeletions.^2,8-11^ In contrast, *GATA4* sequence variants are a rare cause of CDH.^2,12^

## Case Report

**Figure 1.**
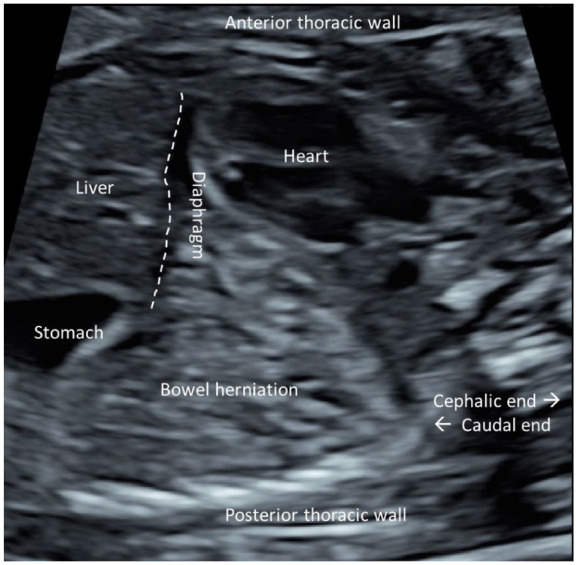
Parasagittal view of the fetus showing the heart dislocated anteriorly to the right side of the thorax due to diaphragmatic herniation of the small bowel to the thoracic cavity posteriorly in the morphological ultrasound image of a 21-week-old fetus. The localisation of the diaphragm is illustrated with a dashed line.

A 29-year-old healthy G2P0 was referred to the Turku University Hospital Fetal Medicine unit due to dextroposition of the fetal heart detected in the morphological ultrasound screening at 20 + 3 weeks of gestation. The first-trimester ultrasound screening and non-invasive prenatal testing (NIPT) had been normal at 12 + 1 weeks. At 21 + 1 weeks of gestation, the heart was situated to the right in the thorax, with the heart axis pointed to the left. Four-chamber and three-vessel views were detected normally. VSD was suspected in the outflow area. The aortic and ductal arches appeared normal. The diaphragm was identified, and the stomach was located within the abdominal cavity. There was a hyperechogenic, non-cystic, space-occupying process in the thorax’s left side, compressing the heart to the right (Figure 1).

Diaphragmatic hernia was suspected. The patient was sent for an MRI, and an amniotic fluid sample was extracted for exome sequencing.

**Figure 2.**
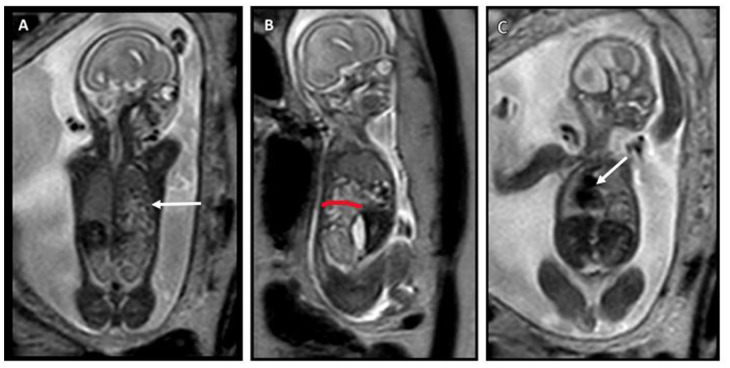
T2-weighted MRI images. (A) A coronal image at the posterior level near the vertebra, showing the left diaphragmatic defect and fluid-filled structures in the thorax (arrow), consistent with bowel loops. (B) A left sagittal image, more clearly depicting the posterior diaphragmatic defect (red line) and bowel in the thoracic cavity. (C) A more anterior coronal image, displaying the rightward shift of the heart and mediastinum (arrow).

MRI was conducted at 21 weeks of gestation and revealed a left posterior diaphragmatic defect, with bowel herniating into the left thoracic cavity (Figure 2). Total fetal lung volume was estimated to be 10.9 ml (−1.3SD for gestational age^13^). The heart and mediastinum were displaced rightwards. No additional abnormalities were identified.

**Figure 3.**
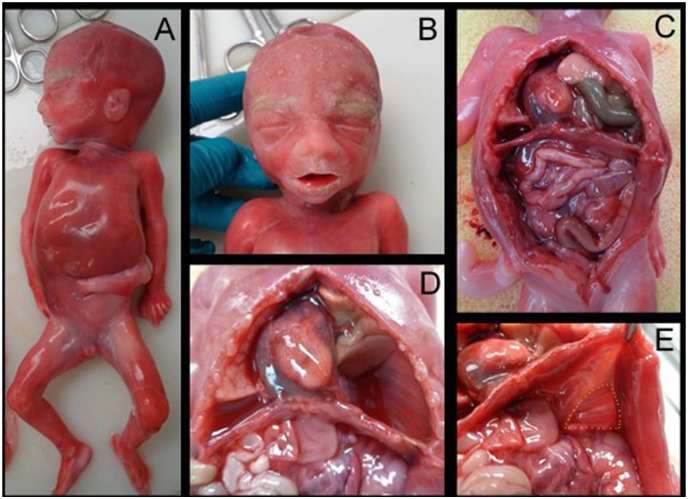
Autopsy photographs. Whole fetus (A) with mild coarseness of facial features (B). The prolapsed intestines and the displacement of the heart to the right side of the thoracic cavity are seen (C). After removing the prolapsed intestines and spleen from the thoracic cavity, the space they were occupying, as well as the displacement of the left lung and heart, could be seen clearly (D). A view of the diaphragm seen from the abdominal cavity (E). The hernia defect is highlighted with yellow dashed lines.

The pregnancy was terminated at 22 + 2 weeks of gestation. Autopsy revealed slight coarseness of facial features and a large posterolateral hernia covering half of the diaphragm’s left side. Most of the intestines and spleen were herniated into the thoracic cavity, displacing the heart to the right and withholding space from the developing lungs. The anatomy and axis of the heart were normal. There were no signs of heart defects. The positioning of the great arteries and veins was normal. (Figure 3). The left lung was significantly smaller than the right lung (right: 7.7 g; left: 3.5 g) (Table S1). Histology revealed slight developmental delay of lung parenchyma: they were in early canalicular phase, in contrast to the expected late canalicular phase. A more detailed description of the macroscopic and microscopic autopsy, with fetal measurements and organ weights, is presented in the Supplemental data.

The Genomics laboratory of Turku University Hospital performed clinical exome sequencing from fetal DNA. A heterozygous nonsense variant c.826C>T,p.(Gln276*) in the *GATA4* gene was discovered. The novel variant occurred *de novo* and was classified as pathogenic according to American College of Medical Genetics and Genomics (ACMG) classification criteria.^14,15^ Detailed description of data analysis and variant interpretation is presented in Supplemental Data.

## Discussion

Screening ultrasound at 20 + 3 weeks of gestation revealed rightward displacement of the fetal heart and a possible VSD. In addition, the left hemithorax showed abnormally echogenic, unidentifiable tissue, which raised suspicion of a diaphragmatic hernia. MRI of the fetus revealed a significant left posterior diaphragmatic hernia, explaining the dextroposition of the heart, which was further confirmed in the autopsy. The internal structures and the chirality of the heart visualised normally in the ultrasound and autopsy. There were no signs of *GATA4-*related congenital heart defects. Given the findings, the prognosis was deemed poor, leading to termination of the pregnancy following consultation with a paediatrician.

Genetic testing revealed a pathogenic *de novo* variant c.826C>T,p.(Gln276*) in the *GATA4* gene. The novel nonsense variant has not been previously reported in the literature or variant databases (ClinVar, HGMD). The variant was classified as pathogenic and undoubtedly explains the fetal phenotype. Pathogenic *GATA4* sequence variants typically cause congenital heart defects.^6^ *GATA4* sequence variants have been reported only in rare instances with diaphragmatic hernia, supporting the notion that diaphragmatic hernia is not a common presentation.^2,12,16^

The ClinVar database lists 32 sequence variants as pathogenic or likely pathogenic in the *GATA4* gene, including nonsense, frameshift and missense variants.^17^ Most of these variants are related to structural congenital heart diseases, and a minority are related to testicular anomalies with or without congenital heart disease, whereas none are directly related to diaphragmatic hernia. However, one pathogenic *GATA4* missense variant has been observed in a patient with congenital left-sided diaphragmatic hernia and septal defects.^16^ Interestingly, this variant is located near the variant c.826C>T reported in our case.

Prenatal CDH cases with *GATA4* variants are rare. Kammoun et al.^18^ reported *GATA4* missense variants causing severe one-sided isolated CDH in two fetuses, with adverse outcomes.^18^ In one family, a small, isolated left-sided hernia was reported in two seemingly asymptomatic *GATA4* carriers. The index patient in the family presented prenatally with isolated CDH, although postnatally with syndromic features including bilateral hydronephrosis, undescended testis, and patent foramen ovale.^12^ In addition, 8p23.1 microdeletions encompassing *GATA4* have been reported prenatally with CDH, congenital heart defects, and growth restriction.^19,20^ Awareness of the whole phenotypic spectrum is needed and can only be improved through accumulating published cases; thus, it is important to report novel findings like our case.

Diaphragmatic hernia has been associated with reduced expression of GATA4,^7^ and CDH related to pathogenic *GATA4* variants is predicted to result from haploinsufficiency through a loss-of-function mechanism.^21^ Functional studies in mice have demonstrated that GATA4 deficiency, especially in mesenchymal-derived muscle connective tissue fibroblasts, causes CDH. Interestingly, both humans and mice with *GATA4* haploinsufficiency present CDH with reduced penetrance.^22^ The incomplete penetrance and variable expressivity reported with *GATA4* variants may explain the relatively low incidence of *GATA4*-related CDH. The complex interplay between genetic, epigenetic and environmental factors may account for the different phenotypic presentations between reported cases. Environmental factors such as maternal vitamin A insufficiency during the first trimester, a known risk factor for CDH, may act as a sensitising factor for the development of CDH following *GATA4* haploinsufficiency.^2,22^

Future research should focus on functional studies of *GATA4* variants to better understand its crucial role during embryogenesis and to understand the complete phenotypic spectrum of *GATA4* variants. Larger cohorts will help in determining the prevalence of *GATA4* variants in CDH cases. This will result in a more comprehensive understanding of the genetic landscape of CDH and its association with *GATA4*. It is recommended that the *GATA4* gene be added to the list of genes of interest for isolated or syndromic prenatal CDH.

## Supplemental Material

sj-docx-1-pdp-10.1177_10935266251381440 – Supplemental material for Prenatal Isolated Congenital Diaphragmatic Hernia: A Rare Clinical Presentation of a GATA4 Pathogenic Variant Supplemental material, sj-docx-1-pdp-10.1177_10935266251381440 for Prenatal Isolated Congenital Diaphragmatic Hernia: A Rare Clinical Presentation of a GATA4 Pathogenic Variant by Nea Tulonen, Jussi Tallus, Heidi Kaprio, Jukka Laine, Mirjami Mattila, Maria Haanpää and Sini Keskinen in Pediatric and Developmental Pathology
