# Supplementary material for: Prenatal Isolated Congenital Diaphragmatic Hernia: A Rare Clinical Presentation of a GATA4 Pathogenic Variant
Source: Pediatr Dev Pathol. 2025 Oct 16;29(1):84–8. doi: 10.1177/10935266251381440 (PMC12779761; doi:10.1177/10935266251381440)
Supplement: sj-docx-1-pdp-10.1177_10935266251381440 – Supplemental material for Prenatal Isolated Congenital Diaphragmatic Hernia: A Rare Clinical Presentation of a GATA4 Pathogenic Variant [file sj-docx-1-pdp-10.1177_10935266251381440.docx]

Supplemental data

Detailed autopsy report

The measurements (weight, crown-rump length, and toe-heel length) of the fetus were consistent with a gestational age of 22 to 23 weeks, matching the estimated gestational age. Facial features had slight coarseness. A large posterolateral hernia of the left side of the diaphragm – approximately half of the left side in size – was detected, with most of the intestines and spleen prolapsed into the thoracic cavity, displacing the heart to the right and withholding space from the developing lungs. The axis and anatomy of the heart and the positioning of the great arteries and veins were normal (Figure 3 of the main article). The macroscopic structure of both lungs was normal, but there was a great difference in size: the left lung weighed 3.5g and the right lung 7.7g (Table S1). Histologically, a slight delay was noted in both lungs as they were in early canalicular phase, in contrast to the expected late canalicular phase. The central nervous system was structurally normal in regard to gestational age, as were the rest of the fetal structures. The placenta, fetal membranes, and the umbilical cord were macroscopically normal; histologically, a variable accumulation of perivillous fibrin was noted in the placenta.

Table S1. The weight and measurements of the fetus, as well as the weights of selected organs, with reference measurements.

| **Autopsy measurements** | **Measurement (reference for gestation age of 22 weeks** ± **1SD^1^)** |
| --- | --- |
| Weight | 512g (438±74) |
| Crown-heel length | 29.5cm (27.5±2.1) |
| Crown-rump length | 20.5cm (18.8±1.6) |
| Toe-heel length | 4.1cm (3.9±0.3) |
| Heart weight | 1.7g (3.3±0.8) |
| Liver weight | 19.8g (20.7±4.3) |
| Brain weight | 79g (63.5±10.5) |
| Lungs’ weight | together 11.2g (11.9±3.5); right 7.7g, left 3.5g |
| Kidneys’ weight | 12.3g (4.2±1.3) |

Results

The fetus presented with a *de novo* heterozygous nonsense variant c.826C>T,p.(Gln276*) in the *GATA4* gene. The variant creates a premature stop codon in exon 4 and a defective or absent protein product, ultimately leading to loss-of-function of the GATA4 protein product. The discovered *GATA4* variant had not, at the time of writing, been reported in the Genome Aggregation Database (gnomAD, v4.1.0), the Human Gene Mutation Database (HGMD), nor the Clinical Variant Database (ClinVar). The variant was absent from both parents. For these reasons, the rare c.826C>T variant was classified as pathogenic according to American College of Medical Genetics and Genomics (ACMG) classification criteria (ACMG class 5: PVS1, PM2).^2,3^

Methods

MRI, conducted at 21 weeks of gestation, was performed using a 1.5 T scanner (Philips Ingenia Ambition X, Philips, Best, NL) and consisted of T2-weighted images in multiple orientations, T1-weighted images, and diffusion-weighted images.

For clinical exome sequencing, DNA was isolated from amniotic fluid using phenol-chloroform extraction. A total of 5,500 clinically significant genes were sequenced by Next-Generation Sequencing (NGS), including 20 bp of 5’ and 3’ intronic regions. Sequencing libraries were generated using SOPHiA Genetics™ Clinical Exome Solution v3 and sequenced with the NextSeq sequencer (Illumina) using 2x151 bp paired-end sequencing technology. Bioinformatics was performed using the Sophia DDM program (v5.10.48, Sophia Genetics). The reference genome used was GRCh38/hg38.

Variant classification was conducted according to ACMG classification guidance.^2,3^ The detected pathogenic variant was reported according to Human Genome Variation Society (HGVS) standard guidance. No other findings of clinical significance were found by NGS analysis. The reference sequence for *GATA4* was NM_001308093.3.

Limitations of the method used include repeat sequence annotation, genomic rearrangements such as translocations, copy number variants smaller than 2 exons, and deep intronic variants. Pseudogenes and regions with segmental duplications might not be reliably analysed with this method.

*Statements and Declarations*

Ethical considerations

All participants provided written informed consent prior to enrolment in the study. Our institution (Turku University Hospital) does not require ethical approval for reporting individual cases or case series.

Consent to participate

A written consent was obtained from the family.

Data availability statement

Data is available upon reasonable request.

References

1. Gilbert-Barness E. and Debich-Spicer D.E.: Handbook of Pediatric Autopsy Pathology, p.71. Humana Press 2005.

2. Kalia SS, Adelman K, Bale SJ et al. Recommendations for reporting of secondary findings in clinical exome and genome sequencing, 2016 update (ACMG SF v2.0): a policy statement of the American College of Medical Genetics and Genomics. Genet Med 2017 192. 2016 Nov;19(2):249–55.

3. Richards S, Aziz N, Bale S et al. Standards and Guidelines for the Interpretation of Sequence Variants: A Joint Consensus Recommendation of the American College of Medical Genetics and Genomics and the Association for Molecular Pathology. Genet Med [Internet]. 2015 May 8 [cited 2024 Sep 12];17(5):405. Available from: /pmc/articles/PMC4544753/
